# Determination of the Membrane Topology of the Small EF-Hand Ca^2+^-Sensing Proteins CaBP7 and CaBP8

**DOI:** 10.1371/journal.pone.0017853

**Published:** 2011-03-22

**Authors:** Hannah V. McCue, Robert D. Burgoyne, Lee P. Haynes

**Affiliations:** Department of Cellular and Molecular Physiology, University of Liverpool, Liverpool, United Kingdom; University of Oldenburg, Germany

## Abstract

The CaBPs represent a subfamily of small EF-hand containing calcium (Ca^2+^)-sensing proteins related to calmodulin that regulate key ion channels in the mammalian nervous system. In a recent bioinformatic analyses we determined that CaBP7 and CaBP8 form an evolutionarily distinct branch within the CaBPs (also known as the calneurons) a finding that is consistent with earlier observations characterising a putative C-terminal transmembrane (TM) spanning helix in each of these proteins which is essential for their sub-cellular targeting to the Golgi apparatus and constitutive secretory vesicles. The C-terminal position of the predicted TM-helix suggests that CaBP7 and CaBP8 could be processed in a manner analogous to tail-anchored integral membrane proteins which exhibit the ability to insert across membranes post-translationally. In this study we have investigated the topology of CaBP7 and CaBP8 within cellular membranes through a combination of trypsin protection and epitope accessibility analyses. Our results indicate that the TM-helices of CaBP7 and CaBP8 insert fully across membranes such that their extreme C-termini are luminal. The observed type-II membrane topology is consistent with processing of CaBP7 and CaBP8 as true tail-anchored proteins. This targeting mechanism is distinct from any other calmodulin related Ca^2+^-sensor and conceivably underpins unique physiological functions of these proteins.

## Introduction

The calcium ion (Ca^2+^) is a signalling intermediate fundamental to many aspects of mammalian physiology and influences processes ranging from fertilisation to cell death, modulation of gene transcription, ion channel function, exocytosis and phospholipid metabolism [1]. Ca^2+^ is able to exert such far reaching effects due to the existence of a diverse complement of dedicated Ca^2+^-sensing proteins each of which exhibits unique Ca^2+^-binding properties coupled to specific patterns of tissue and cellular expression [2]. Ca^2+^-sensors characteristically respond to the magnitude, timing and spatial localisation of a given Ca^2+^ signal to elicit an appropriate intracellular response and subsequent alteration in cellular activity [3]. A key feature of Ca^2+^-sensing proteins is that they often exhibit restricted sub-cellular localisations, a quality fundamental to their ability to integrate specific subsets of Ca^2+^ signals that are in turn linked to particular cellular pathways. The largest group of Ca^2+^-sensors are those comprising the calmodulin (CaM) super-family of small EF-hand containing proteins and members of this family exhibit extensive diversity in both how they bind Ca^2+^ and Mg^2+^ ions and specifically target to unique sub-cellular domains [2]. The Ca^2+^-binding proteins (CaBPs) are a subfamily of the CaM related proteins [4], [5], [6] which have emerged as important regulators of pivotal ion channels and intracellular trafficking enzymes. The CaBPs consist of seven proteins [6] most of which have been demonstrated to regulate specific target proteins and this functional diversity is mirrored by differences in how each CaBP is targeted to discrete sub-cellular sites. CaBP1 has three splice variants [7], two of which become *N*-myristoylated, a post-translational acylation modification essential to membrane association with the Golgi complex and plasma membrane [8], [9]. This targeting mechanism is shared by other Ca^2+^-sensing proteins and has been elaborated on extensively during evolution to provide a surprisingly diverse and flexible means of associating proteins with specific membrane domains [8], [10]. In all instances thus far examined myristoylation of the Ca^2+^-sensor and hence correct sub-cellular targeting has been proven indispensible to protein function [9], [11]. Other members of the CaBP family (Caldendrin, CaBP4 and CaBP5) contain no obvious consensus motifs for myristoylation or other specific targeting information and consistent with this are predominantly cytosolic [12], [13], [14], [15]. It is likely however that they have the capacity to become specifically targeted in response to Ca^2+^-binding through interactions with proteins that themselves specifically localise [16].

Within the CaBP family there exists an additional example of evolutionary diversification with regards to sub-cellular protein targeting in the form of CaBP7 and CaBP8 (also referred to as calneurons II and I respectively, [5]). These proteins contain no consensus motifs for *N*-myristoylation yet display distinct patterns of membrane association when expressed in cells where they are restricted to the trans-Golgi network (TGN) and vesicular structures. The sub-cellular targeting of CaBP7 and CaBP8 is attributable to the presence of a highly hydrophobic helix at the extreme C-terminus of the protein that is predicted to be a transmembrane (TM) domain. In a previous study [15] we analysed the properties of this targeting mechanism and showed that the predicted TM domain was essential for the correct localisation of CaBP7 and CaBP8 to membranes and moreover that this sequence alone was sufficient to target a normally cytosolic protein to identical membrane domains. The C-terminal position of the CaBP7 and CaBP8 TM helix parallels the organisation observed for the tail-anchored (TA) class of integral membrane proteins [17], [18]. This membrane protein type is evolutionarily conserved and includes proteins essential to intracellular trafficking and mitochondrial architecture/function. TA protein topology consists of a large cytosolic N-terminal functional domain, a TM helix and short luminal C-terminus. The key feature of TA proteins is that due to the lack of an N-terminal signal peptide and emergence of their TM domain close to the point of translation termination, they are not typically co-translationally translocated across membranes in a Sec61 dependent manner. Instead it has been observed that TA proteins are dealt with quite differently to other TM proteins and can be inserted across membranes post-translationally although the mechanistic details of how this happens remain to be resolved at the molecular level [19].

In a previous study we described a putative TM helix present in CaBP7 and CaBP8 that could account for correct sub-cellular targeting of both proteins but we did not determine that this was a true TM domain [15]. In the present study we have now formally established that these hydrophobic sequences are indeed TM domains by determining the membrane topology of CaBP7 and CaBP8 through a combination of protease protection [20] and epitope accessibility analyses [21]. Our data indicate that both fluorescently- and myc-tagged reporter constructs behave identically and exhibit a type-II membrane topology. These findings demonstrate unequivocally the presence of functional TM domains in CaBP7 and CaBP8 that are responsible for driving the correct membrane localisation and membrane orientation of the Ca^2+^-sensors. We additionally demonstrate that these proteins are a new TA class of Ca^2+^-sensor. This targeting mechanism is likely to be essential for the emerging function of these proteins as regulators of vesicular trafficking events in the secretory pathway through the control of phosphoinositide metabolism at the TGN [22].

## Materials and Methods

### Molecular biology

N-terminally and C-terminally mCherry or EYFP tagged full length CaBP7 and CaBP8 were generated as previously described [15]. Enhanced cyan fluorescent protein vector (ECFP-C1) was obtained from Clontech (CA, USA). CaBP7 tagged with c-myc at the carboxy-terminal end (mCherry-CaBP7-myc) was made by PCR sub-cloning using wild-type CaBP7 template and previously described sense primer [15] in combination with a reverse primer encoding the ten amino acid sequence (EQKLISEEDL) of c-myc [23]: 5′-AACCAGGTGCTGCGCAGTGGCATGAAGGAACAAAAACTTATTTCTGAAGAAGATCTGTAGCCGCGGGCCCGGATAT-3′. The resultant PCR product was digested with *HindIII* and *SacII* and ligated into mCherry-N1 (a gift from Dr. R. Tsien, University of California).

### Cell culture and transfection

HeLa cells [24] were maintained in DMEM supplemented with 5% foetal bovine serum, 1% penicillin/streptomycin and 1% non-essential amino acids in a humidified atmosphere of 5% CO_2_/95% air at 37°C. Cells were transiently transfected using Genejuice transfection reagent (Novagen) according to the manufacturer's protocol. For co-localisation studies cells plated onto glass coverslips were transfected with 1 µg of each construct.

### Biochemical protease protection assay

Equal numbers of HeLa cells were grown on 6-well plates (Corning) and wells transfected in duplicate with 3 µg of either: mCherry-C1, mCherry-CaBP7 or CaBP7-mCherry. Twenty-four hours post-transfection cells from 1 well for each condition were washed in KHM buffer (110 mM potassium acetate, 20 mM HEPES (pH 7.2), 2 mM MgCl_2_) then treated with 40 µM Digitonin in KHM buffer for 1 min at room temperature. After permeabilisation cells were washed in KHM buffer and challenged with 4 mM trypsin in KHM buffer for 5 min at room temperature. Cells were washed and protein extracted by boiling in an appropriate volume of Laemmli buffer. Control cells not treated with digitonin or trypsin were processed identically. Equal aliquots of the samples were subjected to SDS-PAGE and western blot analysis using a rabbit polyclonal RFP antibody (a gift from Dr. Ian Prior, University of Liverpool). Relative amounts of protein in individual tracks were quanitated using the densitometry function of ImageJ (NIH, USA) and averaged data from multiple independent experiments calculated ± Standard Error of the Mean (SEM).

### Fluorescence protease protection assay

Cells were plated onto glass bottomed dishes (MatTek corporation) and triply transfected with 1 µg of each of the three constructs: ECFP-C1, EYFP-CaBP7/CaBP8 and CaBP7/CaBP8-mCherry. Fluorescence protease protection assays were performed as previously described [25]. Briefly, twenty-four hours after transfection cells were washed three times in KHM buffer at room temperature. The cell chamber was set up on the microscope stage and imaging started during this ‘pre-permeabilisation’ stage. Frames were collected at a rate of 1frame every 8 seconds. Cells were then perfused with KHM buffer containing 40 µM digitonin until ECFP fluorescence started to dissipate. Perfusion was first switched to KHM buffer until all the ECFP fluorescence had disappeared (10–60 sec) and then to KHM containing 4 mM trypsin. Images were recorded until EYFP signal was lost.

### Antibody accessibility experiments

Cells were plated onto glass coverslips and transfected with 1 µg of mCherry-CaBP7-myc or mCherry-CaBP8-myc. 24 hours post-transfection cells were washed and permeabilised in the following ways: Selective permeabilisation of the plasma membrane was obtained by treating cells with 40 µM digitonin in KHM buffer for one minute before fixation using 4% formaldehyde in PBS. Control cells were fixed and permeabilised with 0.2% (v/v) Triton-X-100 for non-selective solubilisation of all cellular membranes. Cells from both conditions were immunostained with either a monoclonal anti-c-myc antibody (1∶100, 9E10 clone, Sigma, Poole, UK) or a rabbit polyclonal anti-RFP antibody (1∶100, a kind gift from Dr I. Prior, University of Liverpool) followed by FITC-conjugated goat anti-mouse or goat anti-rabbit secondary antibodies (1∶75, Sigma, Poole, UK) in PBS containing 5% BSA.

### Fluorescence and microscopy and imaging

Endogenous CaBP7 localisation was detected using a rabbit polyclonal anti-CaBP7 antibody (1∶200, Santa Cruz Biotechnology) followed by staining with FITC conjugated goat anti-rabbit secondary antibody. Fixed and live cells were imaged using a Leica TCS-140 SP-MP microscope (Leica Microsystems, Heidelberg, Germany) with a 22 µm pinhole and x63 oil immersion objective. For multichannel imaging, each channel was imaged sequentially to eliminate bleed-through between channels. Fluorescence intensity was quantified using Leica Lite confocal software (Leica Microsystems) by drawing a region of interest around the Golgi using the stack profile tool. Data was exported into Microsoft Excel (Microsoft Office, 2007) and fluorescence intensity in each frame (F) expressed as a percentage of the fluorescence at the start of the experiment (F_0_). Graphs were generated using Excel and OriginPro 8 (OriginLab corporation). Images were exported as TIFF files and compiled using ImageJ and CorelDraw X4 (Corel Corporation).

## Results

### Localisation of tagged CaBP7 and CaBP8 in HeLa cells

**Figure 1:**
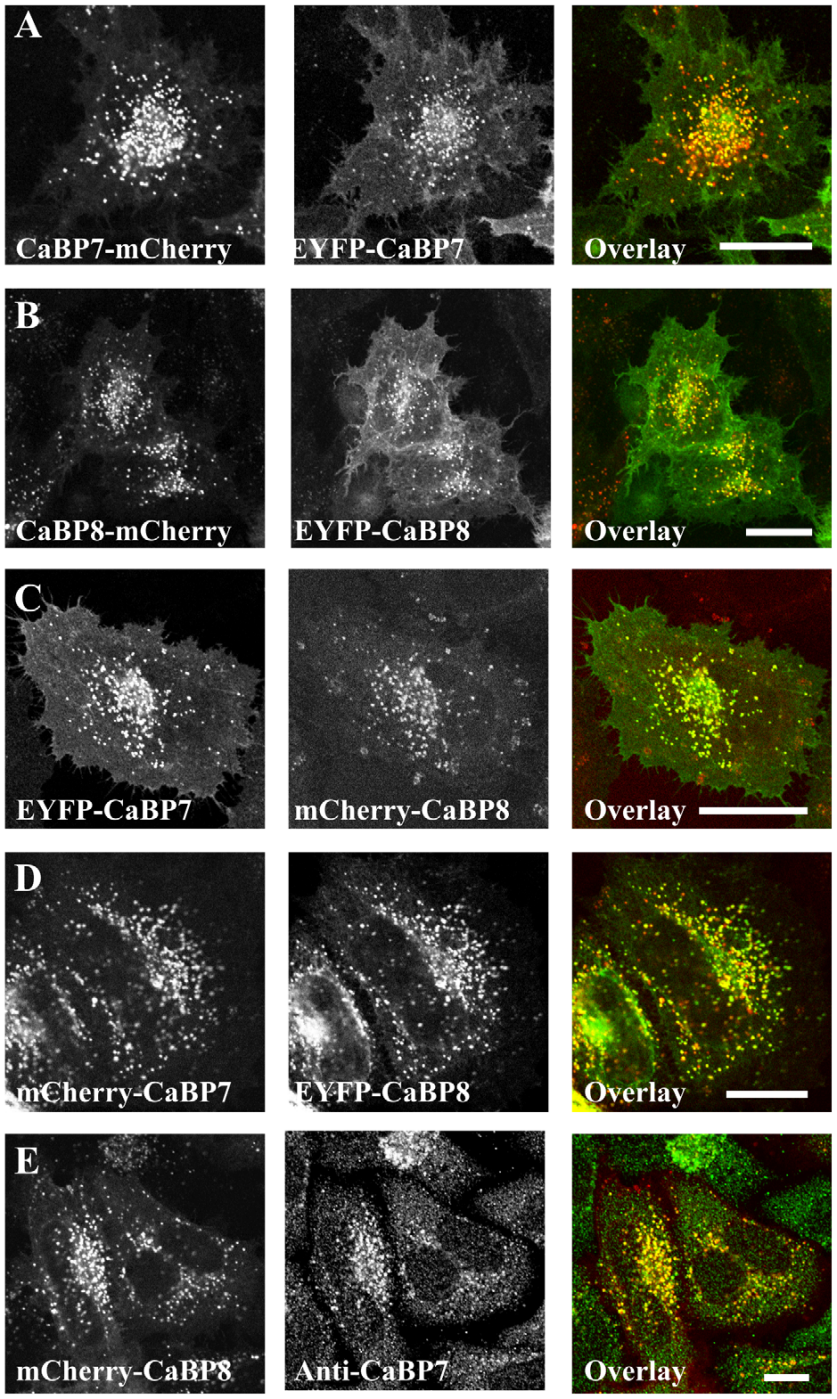
Localisation of fluorescently tagged CaBP7 and CaBP8 in HeLa cells. *A* - Exogenously expressed C-terminally tagged CaBP7-mCherry (red) and N-terminally tagged EYFP-CaBP7 (green). Regions of co-localisation appear yellow in the overlay image. *B* - Exogenously expressed C-terminally tagged CaBP8-mCherry (red) and N-terminally tagged EYFP-CaBP8 (green). Regions of co-localisation appear yellow in the overlay image. *C and D* - Exogenously co-expressed N-terminally EYFP (green) or mCherry (red) tagged CaBP7 and CaBP8. Co-localisation appears as yellow in overlay images. *E* - Exogenously expressed N-terminally tagged mCherry-CaBP8 (red) and immunostained endogenous CaBP7 (Anti-CaBP7, green) in HeLa cells. Co-localisation appears yellow in the overlay image. Scale bars  = 10 µm.

In order to characterise the membrane topology of CaBP7 and CaBP8 we constructed N- and C-terminal fluorescently tagged variants of both proteins and assessed their distributions in transiently transfected HeLa cells (Figure 1). Consistent with our previous studies examining the membrane targeting of these proteins in Neuro2A cells [15], C-terminally tagged CaBP7 and CaBP8 (CaBP7-mCherry and CaBP8-mCherry, Figure 1A & B) localised to vesicular structures throughout the cytosol which appeared to cluster in a peri-nuclear zone including the TGN [15]. Importantly for this study N-terminally tagged variants of the same proteins (EYFP-CaBP7 and EYFP-CaBP8, Figure 1A & B) co-localised extensively with their C-terminally tagged counterparts (Figure 1A & B overlay) indicating that membrane association was unaffected by the position of the fluorescent protein tag. We extended our basic characterisation of the constructs employed in this study by examining the localisations of co-expressed CaBP7 and CaBP8 in HeLa cells (Figure 1C & D). From these analyses we concluded that both CaBP7 and CaBP8 co-localised extensively on the same vesicular structures and that the nature of the fluorescent protein tag did not interfere with normal targeting of either protein (Figure 1C & D). We were also able to validate the correct targeting of fluorescently tagged CaBP8 in HeLa cells by examination of co-localisation with endogenous CaBP7 (Figure 1E). HeLa cells express CaBP7 endogenously as determined by immunofluorescence staining with a CaBP7 specific antibody (Figure 1E, Anti-CaBP7 and western blotting of HeLa cell lysate with the same antibody, data not shown). The observed immunostaining pattern for endogenous CaBP7 matched that observed with tagged exogenous CaBP7 and CaBP8 and displayed localisation to numerous cytosolic puncta with a concentration of vesicular structures in a peri-nuclear region. Importantly, in HeLa cells over-expressing mCherry-tagged CaBP8 (Figure 1E), the tagged protein displayed extensive colocalisation with endogenous CaBP7. Collectively these data show that fluorescent protein tagging of CaBP7 or CaBP8 either N- or C-terminally with mCherry or EYFP has no impact on normal protein localisation as assessed by comparison and colocalisation with endogenous CaBP7.

### Trypsin protection analysis of tagged CaBP7 and CaBP8

**Figure 2:**
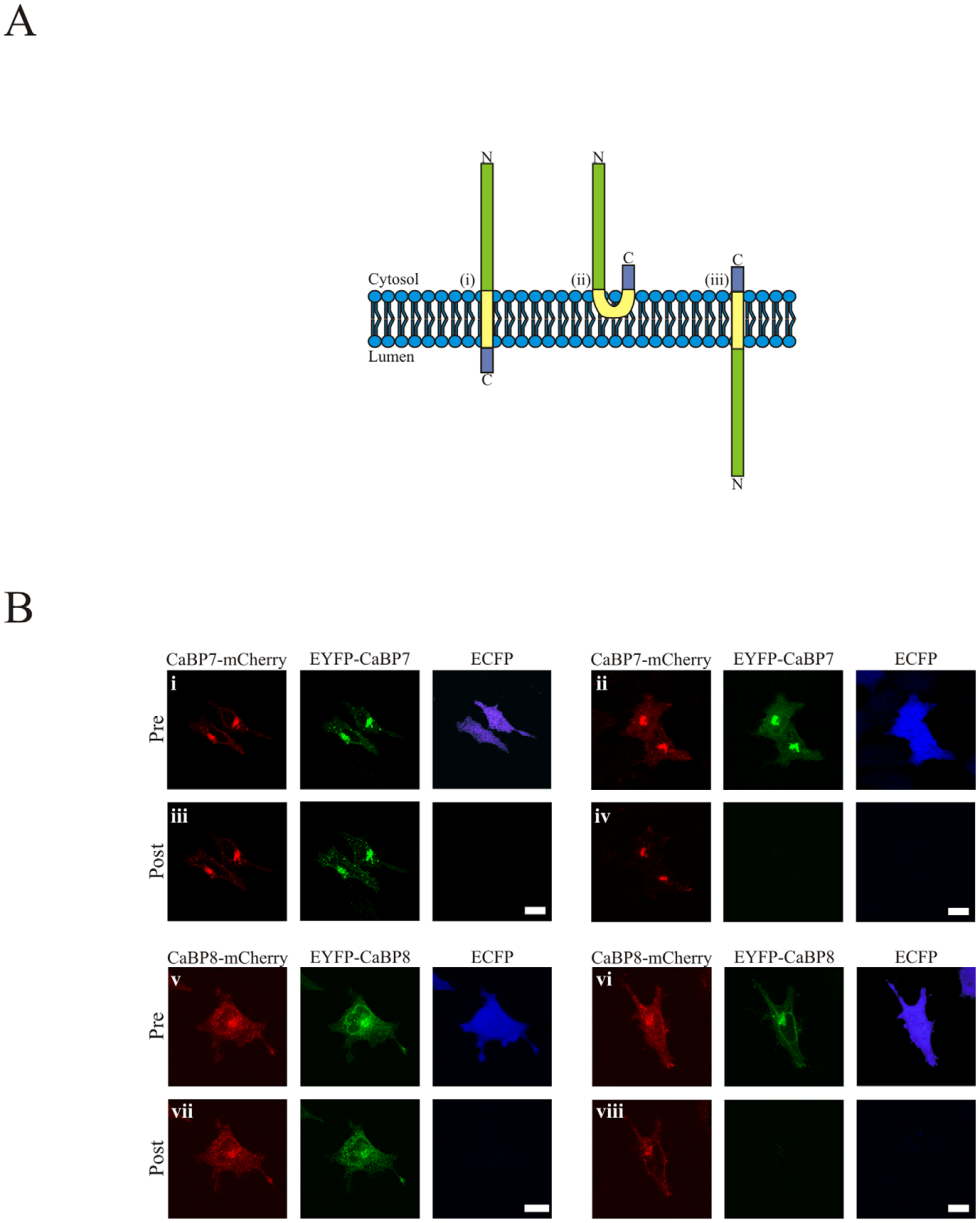
Possible CaBP7 and CaBP8 topologies and representative fluorescence protease protection assay images. *A* – Potential membrane topologies for CaBP7 and CaBP8 across biological membranes: (i) Type II membrane protein orientation; (ii) Peripheral membrane protein orientation; (iii) Type I membrane protein orientation. Protein domains are: Green – N-terminal domain; Yellow – Transmembrane domain and Purple – C-terminal domain. The lipid bilayer is shown in blue. *B* – HeLa cells transfected with ECFP (blue) and N- and C-terminally tagged: CaBP7-mCherry (red) + EYFP-CaBP7 (green) or CaBP8-mCherry (red) + EYFP-CaBP8 (green). ‘Pre’ denotes before digitonin application; ‘Post’ denotes following digitonin and ± trypsin treatment. Controls before (i and v) and after (iii and vii) digitonin but without trypsin treatment for CaBP7 and CaBP8 respectively. Experiments prior to digitonin application (ii and vi) and following both digitonin and trypsin treatment (iv and viii) for CaBP7 and CaBP8 respectively. Scale bars  = 10 µm.

**Figure 3:**
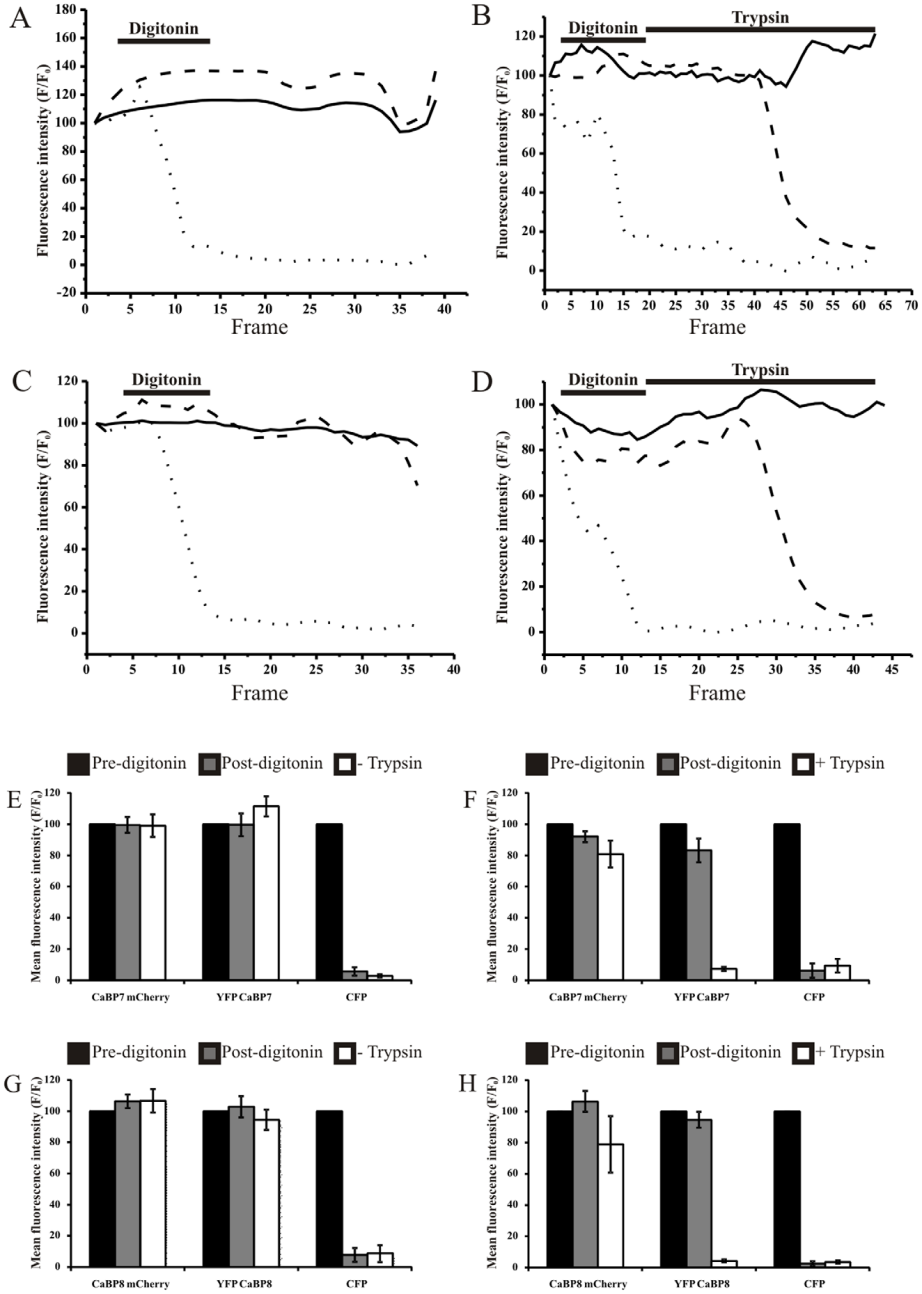
Example fitted curves from individual fluorescence protease protection assays. *A and C* - Control experiments without trypsin treatment for CaBP7 and CaBP8 respectively. *B and D* - CaBP7 and CaBP8 treated with both digitonin and trypsin. Solid lines represent C-terminally mCherry tagged CaBP7 or CaBP8, dashed lines represent N-terminally EYFP tagged CaBP7 or CaBP8 and dotted lines represent ECFP fluorescence. Frame rate  = 1 frame/8 sec. *Histograms for averaged fluorescence protease protection assay results*. *E and G* - Control experiments without trypsin treatment for CaBP7 and CaBP8 respectively. *F and H* - CaBP7 and CaBP8 treated with both digitonin and trypsin. Key: Black  =  before treatment with digitonin, Grey  =  after treatment with digitonin, White (*Controls E and G*)  =  End of experiment with no trypsin treatment, White (*F and H*)  =  End of experiment post-trypsin treatment. (E) n = 7 cells, (F) n = 12 cells, (G) n = 7 cells, (H) n = 7 cells. Data are plotted as mean ± SEM.

CaBP7 and CaBP8, at the primary sequence level, have architectures similar to documented TA proteins. The likely topology for CaBP7 and CaBP8 at organelle membranes would therefore correspond to a cytosolically oriented N-terminus, membrane spanning TM domain and short luminal C-terminus (Figure 2Ai). This topology, although predicted, required formal confirmation as alternative arrangements including: 1) N- and C-termini facing the cytosol with the TM helix only partially inserting into the membrane (Figure 2Aii) or 2) N-terminus luminal with C-terminus cytosolic (Figure 2Aiii) were also valid possibilities. Distinguishing between these topologies represented an important task as it would firstly indicate if CaBP7 and CaBP8 shared TA class protein organisation and secondly it would provide a topological basis for published functional effects of CaBP7 and CaBP8 in the regulation of a cytosolic Golgi associated enzyme, phosphatidylinositol 4-kinaseIIIβ (PI4K, [22]). In these assays we employed an imaging based protocol whereby HeLa cells were triply transfected with ECFP protein and CaBP7 or CaBP8 tagged individually at the N-terminus with EYFP and at the C-terminus with mCherry. This approach was based on a well characterised method which had previously been employed to study the topology of membrane proteins resident on various cellular organelles including the Golgi apparatus [20]. Digitonin was applied to cells and plasma membrane permeabilisation monitored by loss of soluble cytosolic ECFP. Perfusion media was then switched to one containing trypsin which now had cytosolic access and mCherry and EYFP fluorescence signals at the TGN monitored over time. From Figure 2A it can be reasoned that if the topology matched that depicted in (i) then an N-terminal fluorescent tag signal should be eliminated on trypsin application with a C-terminal tag afforded protection inside the lumen of the TGN. If the topology were that depicted in Figure 2A(ii) then both N- and C-terminal fluorescent tag signals would disappear on trypsin treatment. Finally, if the protein topology were that depicted in Figure 2A(iii) then only the C-terminal fluorescent tag signal would be sensitive to the presence of trypsin. Control experiments were first established whereby cells were perfused with KHM buffer until a steady fluorescence baseline had been achieved (Figure 2B(i), CaBP7 and Figure 2B(v), CaBP8). Digitonin was applied but cells were not subsequently challenged with trypsin (Figure 2B(iii), CaBP7 and Figure 2B(vii), CaBP8). The plasma membrane had been effectively compromised in these cells as determined by loss of ECFP signal on digitonin treatment (ECFP, Figure 2B (i & iii, CaBP7) and Figure 2B (v & vii, CaBP8)) however in the absence of trypsin, as expected, no significant loss of mCherry or EYFP fluorescence was observed at the TGN in these cells (Figure 2B and representative fluorescence intensity traces Figure 3A (CaBP7) and Figure 3C (CaBP8)).

In cells treated with digitonin and then challenged with trypsin there was a time dependent loss of EYFP signal in both CaBP7 and CaBP8 expressing cells (Figure 2B(ii), CaBP7 pre-digitonin/trypsin versus Figure 2B(iv), CaBP7 post-digitonin/trypsin and Figure 2B(vi), CaBP8 pre-digitonin/trypsin versus Figure 2B(viii), CaBP8 post-digitonin/trypsin). In contrast mCherry tagged CaBP7 and CaBP8 at the TGN were protected from proteolysis (Figure 2B and representative fluorescence intensity traces Figure 3B (CaBP7) and Figure 3D (CaBP8)). Results obtained from multiple independent experiments were averaged (Figure 3E-H) and clearly demonstrated specific protection of the mCherry fluorescent tag in the presence of trypsin (Figure 3F (CaBP7) and Figure 3H (CaBP8)). Since EYFP was attached to the N-terminus of CaBP7 or CaBP8 whereas mCherry was attached at the C-terminus of both proteins these data are fully consistent with an N-terminal cytosolic/C-terminal lumenal topology (Figure 2Ai).

**Figure 4:**
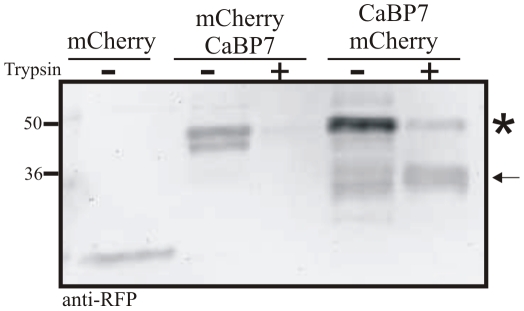
Biochemical protease protection assay. HeLa cells expressing mCherry control protein, mCherry-CaBP7 or CaBP7-mCherry were digitonin permabilised followed by incubation ± trypsin. Cells were lysed, protein resolved on SDS-PAGE and mCherry detected by immunoblotting with polyclonal anti-RFP antibody. (*) - Denotes full length mCherry-CaBP7 or CaBP7-mCherry; (←) - Denotes the major proteolytic fragment generated from CaBP7-mCherry following trypsin digestion. Molecular weight standards (kDa) are shown on the left side of the image.

In related experiments, HeLa cells were transfected with CaBP7 tagged at either the N- or C-terminus with mCherry and processed in the same manner as described above. In these analyses cellular protein was instead monitored by western blotting with mCherry specific antibody (Figure 4). In control cells expressing mCherry alone and having no exposure to trypsin a clear immunoreactive band at ∼26 kDa is observed which corresponded to free mCherry protein (Figure 4, mCherry(−)). In cells expressing N-terminally tagged CaBP7 which were digitonin permeabilised but not trypsin treated an immunoreactive band at ∼50 kDa was observed corresponding to the fusion protein (Figure 4, mCherry-CaBP7 (−) asterisk). Consistent with our imaging studies when these cells were challenged with trypsin all mCherry immunoreactivity was lost (Figure 4, mCherry-CaBP7 (+)) which would be expected if the N-terminal domain of CaBP7 was oriented toward the cytosol. In cells transfected with the C-terminally tagged variant of CaBP7 (CaBP7-mCherry) the full-length fusion protein observed in non-trypsinised cells (Figure 4, CaBP7-mCherry (−) asterisk) was only partially degraded to a major species migrating at ∼30 kDa on SDS-PAGE (Figure 4, CaBP7-mCherry (+) arrow). Using densitometry quantitation we were able to determine that the protein remaining in trypsin treated mCherry-CaBP7 samples was only 25±11.9% (n = 3 independent experiments, ± SEM) of that present in trypsin treated CaBP7-mCherry samples. These results are consistent with trypsin proteolysis of the CaBP7 N-terminus while the TM domain, C-terminus and C-terminal mCherry tag remain protected due to their presence either within the TGN membrane or lumen.

**Figure 5:**
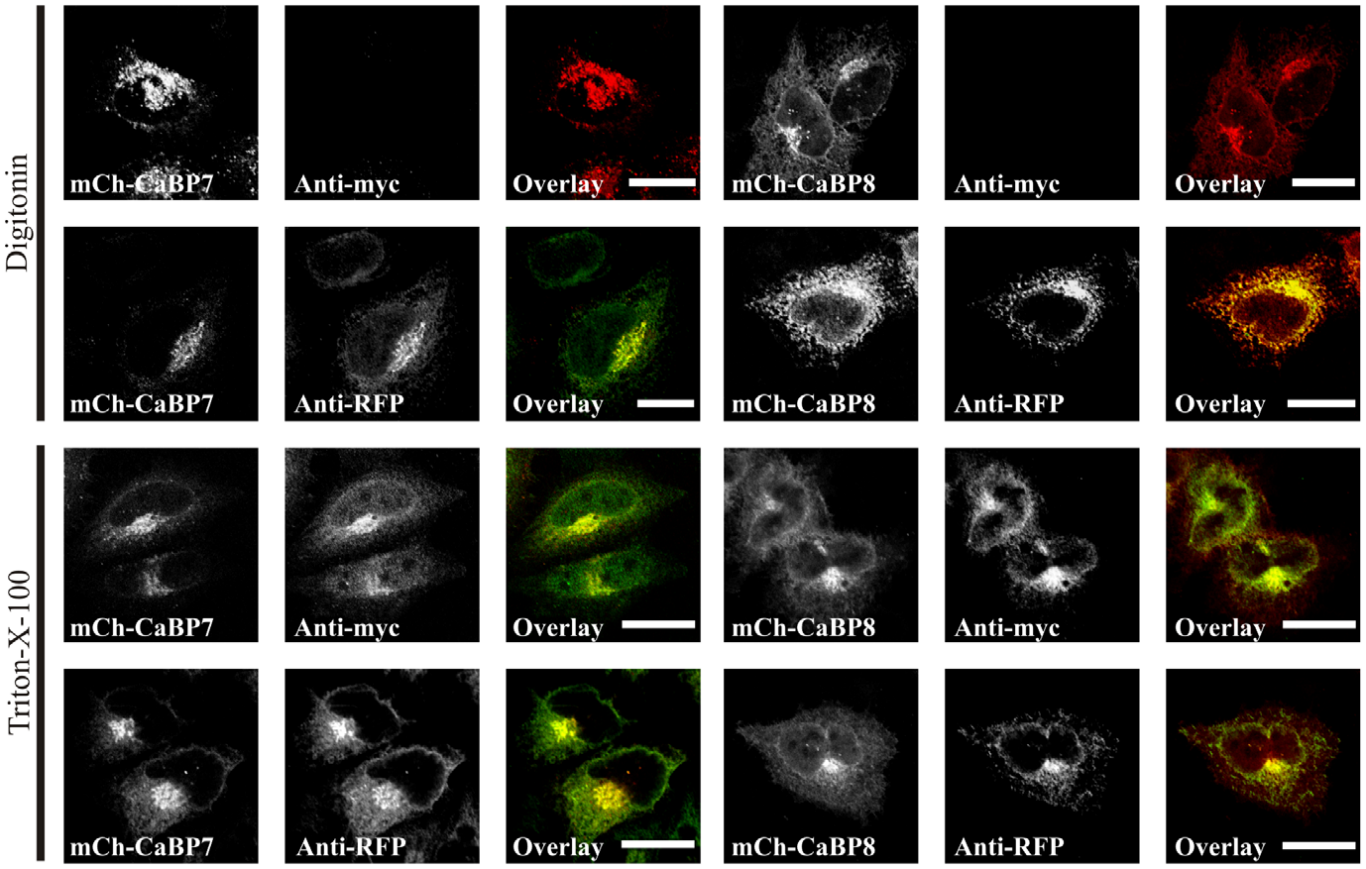
Epitope accessibility analysis of C-terminally myc tagged CaBP7 and CaBP8. *Digitonin permeabilisation -* HeLa cells expressing mCherry-CaBP7-myc or mCherry-CaBP8-myc (mCh-CaBP7-myc and mCh-CaBP8-myc, red) were treated with digitonin to permeabilise the plasma membrane, fixed and processed for immunofluorescence with an anti-c-myc antibody (*A*, mCh-CaBP7-myc and *E*, mCh-CaBP8-myc, Anti-myc, green) or an anti-RFP antibody (*B*, mCh-CaBP7-myc and *F*, mCh-CaBP8-myc, Anti-RFP, green). *Triton-X-100 permeabilisation* - HeLa cells expressing mCh-CaBP7-myc or mCh-CaBP8-myc were fixed, treated with Triton-X-100 to permeabilise all cellular membranes and processed for immunofluorescence with an anti-c-myc antibody (*C*, mCh-CaBP7-myc and *G*, mCh-CaBP8-myc, Anti-myc, green) or an anti-RFP antibody (*D*, mCh-CaBP7-myc and *H*, mCh-CaBP8-myc, Anti-RFP, green). Regions of colocalisation appear yellow in overlay images. Scale bars  = 10 µm.

Since the fusion of peptide tags >25 residues C-terminal to a TM domain has been shown to cause aberrant processing of the resultant chimera as a type-II membrane protein [17], [26], [27] we have extended our analyses to confirm that CaBP7 and CaBP8 can indeed behave in the same manner as true TA class proteins through epitope accessibility analysis of a c-myc tag inserted in the C-terminal tail of the proteins [21] (mCh-CaBP7-myc and mCh-CaBP8-myc, Figure 5). Unlike the C-terminally mCherry tagged proteins which have the potential to be processed as type-II membrane proteins, the C-terminal tail lengths of mCherry-CaBP7-myc and mCh-CaBP8-myc (20 residues) are within the aforementioned experimentally determined limit required for processing as TA proteins. Data derived from these constructs were consistent with our trypsinisation assays and showed that following plasma membrane digitonin permeabilisation and subsequent fixation mCherry fluorescence was present but that no anti-myc immunofluorescence was detectable (Figure 5A, mCh-CaBP7-myc and Figure 5E, mCh-CaBP8-myc). In cells fixed and permeabilised with Triton-X-100 prior to processing for anti-myc immunofluorescence, both mCherry signal and anti-myc immunoreactivity were detectable (Figure 5C, mCh-CaBP7-myc and Figure 5G, mCh-CaBP8-myc). We were additionally able to show that in identically processed cells the mCherry tag was indeed cytosolic through immunostaining with an anti-RFP antibody. Anti-RFP immunoreactivity was detectable in digitonin (Figure 5B, mCh-CaBP7-myc and Figure 5F, mCh-CaBP8-myc) permeabilised cells (in contrast with complete absence of anti-myc immunoreactivity in identically treated cells) confirming the cytosolic orientation of the mCherry tag. Anti-RFP immunoreactivity was unaffected by Triton-X-100 permeabilisation (Figure 5D, mCh-CaBP7-myc and Figure 5H, mCh-CaBP8-myc). Collectively, these data indicate that the C-terminal myc-tags were resident within an internal sub-cellular membrane compartment that was only accessible to anti-myc antibody following Triton permeabilisation of internal membranes.

## Discussion

Signalling pathways controlled by fluctuations in intracellular free Ca^2+^ are central to normal mammalian physiology. The ability of Ca^2+^ to regulate a multitude of cellular activities is fundamentally linked to the expansion and diversification of dedicated Ca^2+^-sensing protein families throughout evolution [2], [3], [28]. Specific spatio-temporal Ca^2+^ signals are detected and integrated by proteins having characteristic sub-cellular localisations, affinities for Ca^2+^-binding and restricted sets of target interactions. The outcome of a given Ca^2+^ signal therefore depends upon the precise nature of the signal (magnitude, timing, location, oscillatory behaviour etc.) and the biophysical properties of available Ca^2+^-sensor proteins. Precisely how Ca^2+^-sensors target to unique sub-cellular locations is therefore key to our understanding of how Ca^2+^ signals are able to influence exclusive target interactions and subsequent changes in cellular activity. Restricting a Ca^2+^-sensor to a particular sub-domain within the cell conceivably allows for target interactions to be influenced over short time scales and in response to tightly localised Ca^2+^-signals. In contrast Ca^2+^-sensors with a higher degree of cellular mobility might couple to broader spatio-temporal Ca^2+^-signals and generate comparatively slower cellular responses [29].

Post-translational *N*-myristoylation as a membrane targeting mechanism in the CaM super-family is well documented and a multitude of evolutionary adaptations centred on this modification have proven functional consequences [8]. CaBP7 and CaBP8 were initially identified based on homology with CaM [5] and in common with CaM contained no consensus motifs for myristoylation. It was somewhat surprising therefore that expression of both of these proteins in cells led to a distinctive pattern of localisation to the TGN and vesicles [15]. We clarified this issue by identifying and characterising a predicted C-terminal membrane helix that mediated the restricted sub-cellular localisations of CaBP7 and CaBP8. Outstanding issues relating to this targeting mechanism concerned firstly whether the putative hydrophobic C-terminal region was indeed a functional TM domain? and secondly, what was the exact topology adopted by both proteins at their target membranes? The C-terminal position of the CaBP7 and CaBP8 TM helix strongly resembles the organisation expected of a classic tail-anchored (TA) protein [18]. This family of proteins typically have a comparatively large cytosol-oriented N-terminal functional domain a single TM spanning domain and short (typically <25 residues) C-terminal luminal domain. TA proteins can therefore be considered a special class of type-II membrane protein and depending on the exact composition of their TM domain are directed either to the endoplasmic reticulum (ER) from where they can traffic on to other destinations along the secretory pathway or are immediately inserted into the mitochondrial outer membrane which represents a trafficking endpoint. In co-localisation studies with mitochondrial markers we have ascertained that CaBP7 and CaBP8 do not traffic to these organelles (unpublished data). TA class proteins represent 2.02% of all coding open reading frames in the human genome [30] and have been implicated in important aspects of cell physiology ranging from control of mitochondrial function, apoptosis and intracellular vesicular trafficking. TA proteins lack a signal peptide and since their TM domain only exits the ribosome near translation termination they are not, in the majority of cases, believed to be co-translationally translocated across the ER membrane in a Sec61 translocon dependent fashion [19]. Various studies have examined the requirement for other protein factors in mediating post-translational insertion of TA proteins into the ER/mitochondrial membranes and at present it appears that at least three distinct pathways are involved in this process, each broadly exhibiting a preference for TM helices of distinct hydrophobicity [19].

We had previously shown that CaBP7 and CaBP8 are membrane associated [15]. In this study we have focused on determining firstly the exact orientation adopted by CaBP7 and CaBP8 at cellular membranes and secondly whether they are processed as TA class proteins. These findings are fundamental as the topologies of CaBP7 and CaBP8 have clear implications for their reported biological activity in the regulation of the essential Golgi trafficking enzyme PI4K which is a cytosolic effector [22]. In order for CaBP7 and CaBP8 to interact with PI4K and respond to fluxes in cytosolic Ca^2+^ their EF-hand containing N-terminal domains, as described, would need to face the cytosol. Their C-termini could be either luminal (if the TM domain passed completely across the lipid bilayer) or cytosolic (if the TM domain only partially integrated into the membrane). It would however be problematic to reconcile published functional data for both proteins if their N-terminal domains were oriented toward the lumen of the TGN. We aimed to distinguish between the various possible topologies through application of a trypsin protection analysis [20] of CaBP7 and CaBP8 tagged with fluorescent protein variants either N- or C-terminally. We demonstrated that only C-terminal tags are afforded protection from cytosolic proteolysis. These data indicate that the C-terminus of both CaBP7 and CaBP8 must reside within the TGN lumen (their TM helix fully inserts across the lipid bilayer) and that they do adopt a cytosolic N-terminal topology required for reported regulation of PI4K. A potential artefact of this assay relates to the fact that TA proteins are a special instance of type-II membrane proteins and it has been documented that appendage of protein tags at the C-terminus of TA-proteins can result in co-translational processing as typical type-II proteins [26], [27]. In the case of the TA protein cytochrome b5 it has been demonstrated that addition of up to 85 residues C-terminal to its TM helix still supports post-translational membrane insertion [31]. Collectively these studies indicate that there may be additional determinants within TA protein sequences that are involved in selection for processing by post-translational mechanisms. To further characterise CaBP7 and CaBP8 membrane insertion we extended our topological analyses to examine accessibility of a ten residue c-myc epitope inserted within the C-terminal tail [21]. This inclusion increased the C-terminal domain length of CaBP7 and CaBP8 to 20 amino acids and therefore they would still be expected to be processed only by dedicated TA protein membrane insertion mechanisms. Our data clearly indicate that the C-terminus of both reporter proteins reside within the lumen of sub-cellular organelles confirming the type-II topology suggested by our trypsin protection assays. That minimal perturbation to the length of the C-terminal tail of CaBP7 and CaBP8 permits a type-II membrane topology suggests post-translational processing by a tail-anchor specific mechanism.

In this study we establish the existence of functional TM domains in CaBP7 and CaBP8 and describe the first example of TA protein targeting in the CaM related super-family of small EF-hand Ca^2+^-sensors. TA proteins are conserved throughout evolution [30] and may represent one of the most primitive membrane protein architectures. Proteins of this class may have evolved prior to functional translocon machineries which would be consistent with their ability to insert into membranes independently of this pathway. Interestingly, the CaBP protein sub-family seems to have appeared at a point in evolution that coincided with the development of vertebrate species [28] an observation that renders the use of TA protein targeting in this family all the more unique. The likely post-translational targeting machinery utilised by CaBP7 and CaBP8 remains to be determined. Based on calculated hydrophobicity scores for the CaBP7 and CaBP8 TM domains of 50.7 and 48.1 respectively [32], [33] it might be speculated that either an Asna-1 [34] or signal-recognition particle [35] mediated pathway is involved.

Within the CaBP family, CaBP1, CaBP7 and CaBP8 have all been shown to target in part to the Golgi apparatus where they perform distinct functions [9], [15], [22]. In the case of CaBP1, targeting relies on N-terminal myristoylation [9] whilst CaBP7 and CaBP8 utilise a C-terminal TM domain that we partially characterised in previous studies [15] and which we formally establish in this report. One question that arises is why are distinct membrane targeting mechanisms required for related Ca^2+^-sensing proteins to associate with the same organelle? Differences in protein function observed at the Golgi between CaBP1 and CaBP7/CaBP8 could be solely due to the existence of specific target-protein interactions mediated by the CaBPs themselves. This interpretation fails to explain our observations regarding diversification in membrane targeting (myristoylation versus TM domain) and it is tempting to speculate that this has a similarly important role to play in protein function perhaps through spatial restriction to membrane micro-domains on the same organelle which could conceivably influence target protein interaction specificity.
